# TGF-β signal rewiring sustains epithelial-mesenchymal transition of circulating tumor cells in prostate cancer xenograft hosts

**DOI:** 10.18632/oncotarget.12808

**Published:** 2016-10-21

**Authors:** Guangcun Huang, Pawel A. Osmulski, Hakim Bouamar, Devalingam Mahalingam, Chun-Lin Lin, Michael A. Liss, Addanki Pratap Kumar, Chun-Liang Chen, Ian M. Thompson, Lu-Zhe Sun, Maria E. Gaczynska, Tim H.-M. Huang

**Affiliations:** ^1^ Departments of Molecular Medicine Cancer Research and Therapy Center and School of Medicine, University of Texas Health Science Center at San Antonio, San Antonio, Texas, USA; ^2^ Departments of Cellular and Structural Biology Cancer Research and Therapy Center and School of Medicine, University of Texas Health Science Center at San Antonio, San Antonio, Texas, USA; ^3^ Departments of Medicine Cancer Research and Therapy Center and School of Medicine, University of Texas Health Science Center at San Antonio, San Antonio, Texas, USA; ^4^ Departments of Urology Cancer Research and Therapy Center and School of Medicine, University of Texas Health Science Center at San Antonio, San Antonio, Texas, USA; ^5^ Departments of Radiation Oncology Cancer Research and Therapy Center and School of Medicine, University of Texas Health Science Center at San Antonio, San Antonio, Texas, USA

**Keywords:** tumor metastasis, circulating tumor cells, epithelial-mesenchymal transition (EMT), transforming growth factor-β (TGF-β), positive feedback signaling

## Abstract

Activation of TGF-β signaling is known to promote epithelial-mesenchymal transition (EMT) for the development of metastatic castration-resistant prostate cancer (mCRPC). To determine whether targeting TGF-β signaling alone is sufficient to mitigate mCRPC, we used the CRISPR/Cas9 genome-editing approach to generate a dominant-negative mutation of the cognate receptor TGFBRII that attenuated TGF-β signaling in mCRPC cells. As a result, the delicate balance of oncogenic homeostasis is perturbed, profoundly uncoupling proliferative and metastatic potential of *TGFBRII*-edited tumor xenografts. This signaling disturbance triggered feedback rewiring by enhancing ERK signaling known to promote EMT-driven metastasis. Circulating tumor cells displaying upregulated EMT genes had elevated biophysical deformity and an increase in interactions with chaperone macrophages for facilitating metastatic extravasation. Treatment with an ERK inhibitor resulted in decreased aggressive features of CRPC cells *in vitro*. Therefore, combined targeting of TGF-β and its backup partner ERK represents an attractive strategy for treating mCRPC patients.

## INTRODUCTION

Prostate cancer is the third most commonly diagnosed cancer in the United States with an estimated 220,800 new cases and 27,540 deaths in 2015 [1]. Androgen-deprivation therapy remains the principal treatment for patients with biochemical recurrence; however, some patients eventually develop metastatic castration-resistant prostate cancer (mCRPC) with malignant lesions detected in distant organs including bones [2]. Additional treatments, such as chemotherapy and target therapy, are needed for CRPC patients. Nevertheless, second-line therapeutics often present discouraging results in patients who later succumb to the disease [3].

The development of mCRPC is multifaceted, and the underlying mechanism remains elusive. Emerging evidence indicates that epithelial-to-mesenchymal transition (EMT) is a critical process to promote cancer invasion and metastasis including mCRPC [4, 5]. Malignant cancer cells migrating to the leading edge of tumors may shed into the bloodstream of prostate cancer patients. We have recently found that some of these circulating tumor cells (CTCs) undergo active EMT and acquire changes in biophysical properties such as increased deformity, elasticity, and surface adhesiveness [6]. These unique biophysical features allow CTCs to survive and extravasate out of blood vessels for metastatic colonization in distant tissues with supporting niches [4, 7]. In contrast, CTCs without these EMT-mediated biophysical changes in cellular deformity and elasticity are less viable in the mechanically challenging environment of the bloodstream and less fitted to extravasate circulation [7]. Therefore, therapeutic agents targeting EMT could provide an important treatment strategy for slowing the development of mCRPC.

Activation of the transforming growth factor-β (TGF-β) pathway and the associated signaling cascade has previously been shown to promote EMT [5, 8, 9]. This prior observation is further supported by our previous finding that TGF-β-driven upregulation of EMT-related genes can be detected in CTCs isolated from mCRPC patients [6]. In this study, we further used the CRISPR/Cas9 genome-editing technique to generate a dominant-negative mutation of the cognate TGF-β receptor type II (TGFBRII) that is capable of attenuating TGF-β signal transduction in castration-resistant DU145 prostate cancer cells. We then determined how deregulation of this signaling homeostasis tips the balance of growth and metastatic potential of tumor xenografts. While the *TGFBRII*-editing resulted in disruption of signaling network traffic for TGF-β-driven EMT, we additionally found that the disturbance triggered a feedback mechanism by activating extracellular signal-regulated kinase (ERK) for supporting EMT-mediated metastasis. This dominant-negative mutation also exerted profound effects on the expression of EMT-related genes and biophysical features of CTCs, as well as their interactions with host macrophages. Altogether, our studies identify an important back-up mechanism of TGF-β-driven EMT in mCRPC. The finding has an important therapeutic implication in that dual blockade of the key and feedback pathways for EMT can be an additional option for treating advanced prostate cancer patients.

## RESULTS

### Genome editing of *TGFBRII* deregulates TGF-β signaling networks that trigger an ERK feedback response

We conducted a comparative study of TGF-β-driven EMT in cultured CRPC cells and in CTCs and the enumeration of CTCs and accompanying host macrophages isolated from xenograft hosts and prostate cancer patients, respectively (Supplementary Figure S1). It is known that aberrant activation of TGF-β signaling up-regulates the expression of transcription factors that promote EMT, contributing in part to a metastatic phenotype in prostate cancer [10, 11]. TGF-β ligands bind to the TGFBRII receptor, followed by recruitment and phosphorylation of another receptor, TGF-β receptor type I (TGFBRI) [12, 13]. Forming a heterodimer, the receptor complex then propagates the signal through interactions with SMAD proteins that are translocated to the nucleus to regulate gene transcription [8, 13]. Upregulation of *TGFBRII* is also linked to poor prognosis of patients with advanced prostate cancer (Supplementary Figure S2). Therefore, TGFBRII is an ideal target for signaling blockade of EMT-mediated metastasis.

**Figure 1:**
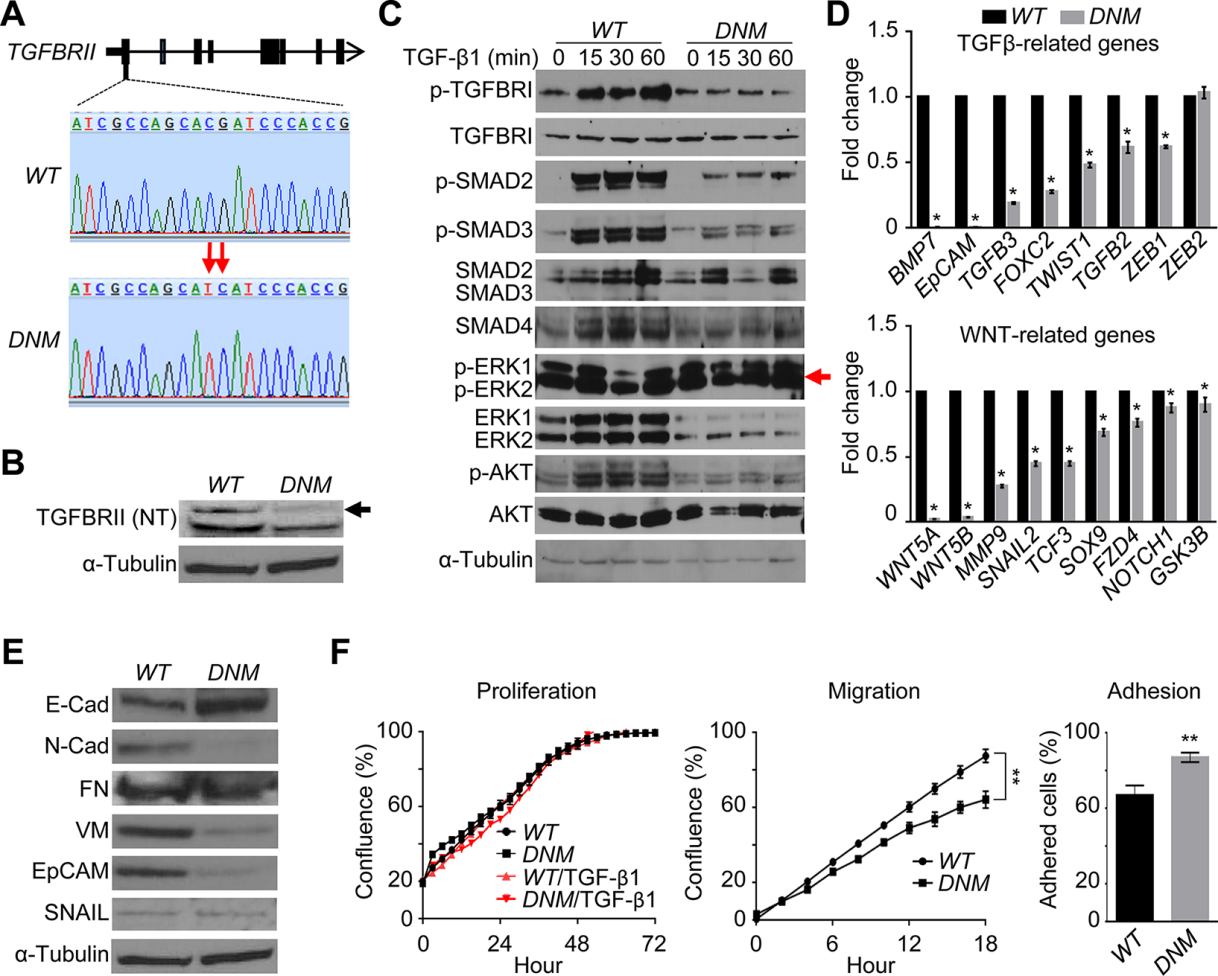
Genome editing of *TGFBRII* disables TGF-β signaling networks and triggers ERK feedback response (**A**) Genomic DNA was extracted from DU145 *TGFBRII* wildtype *(WT)* and lenti TGFBRII-gRNA/Cas9-treated cells, respectively, subjected to PCR amplification, and subsequently purified products were sequenced to confirm *TGFBRII* gene editing (*domain negative mutation* or *DNM*). (**B**) Cellular total proteins were extracted from *WT* and *DNM* cells, respectively, and subjected to immunoblotting against anti-TGFBRII (N-terminal) antibody. (**C**) Cells were treated with or without 5 ng/ml of recombined human TGF- β1 for 0–60 min, and cellular total proteins were extracted and subjected to immunoblotting against different TGF-β signaling pathway players. (**D**) Traditional real-time qPCR of key EMT genes related to TGF-β and WNT signaling pathways in *WT* and *DNM* cells. *p < 0.05. (**E**) Western blot analysis of key EMT proteins in *WT* and *DNM* cells. (**F**) Cell proliferation, migration (wound healing) and adhesion assay, respectively, using IncuCyte^®^ ZOOM live-cell kinetic imaging system. **p < 0.01.

**Figure 2:**
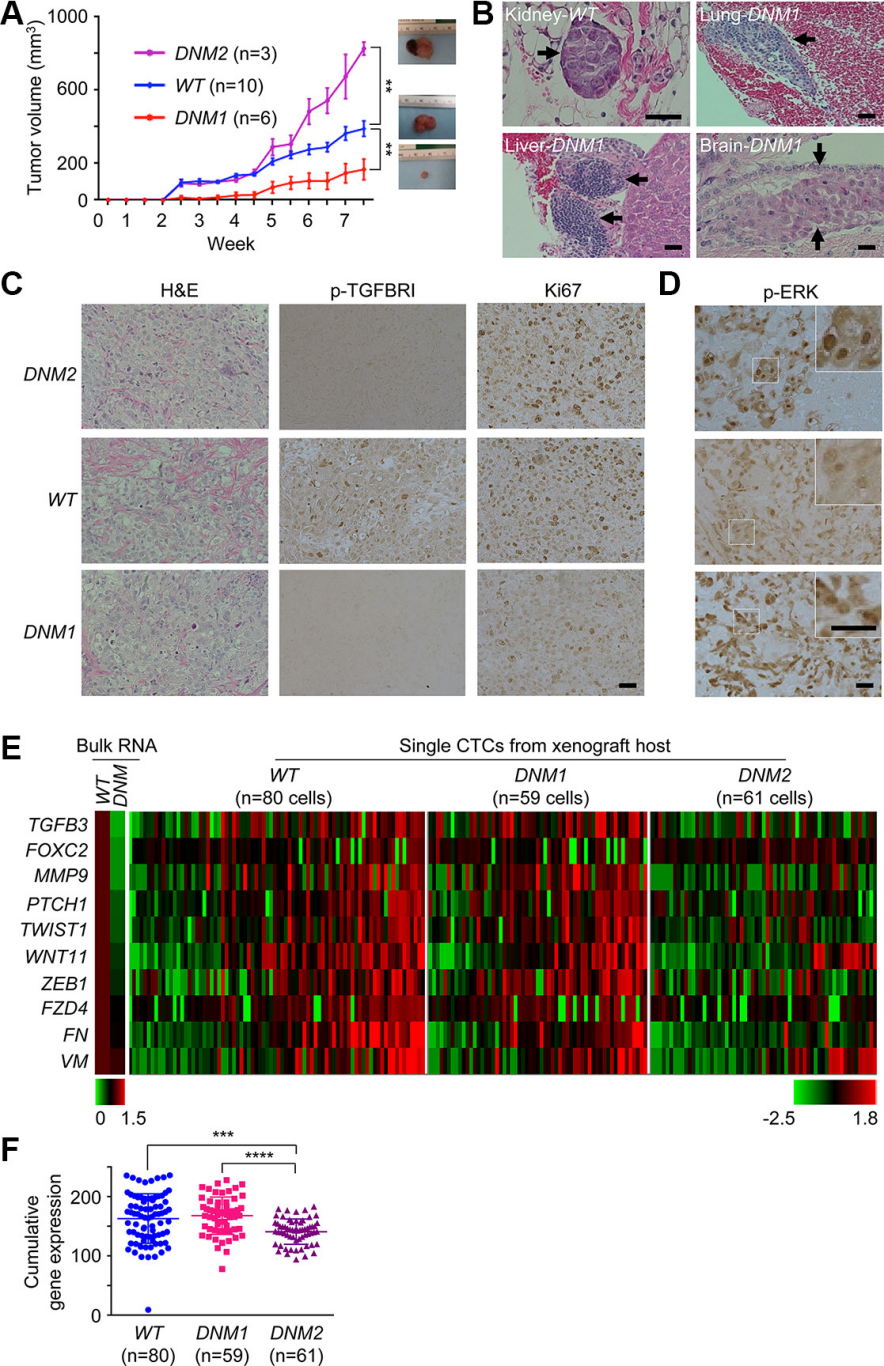
Disabling TGF-β signaling homeostasis leads to uncoupled growth and metastatic potential of tumor xenografts (**A**) Tumor growth curve in DU145 xenograft mouse model. DU145 *TGFBRII WT* and *DNM* cells, respectively, were subcutaneously injected in the flank of 5-week-old male nude mice and xenografts were measured externally in two dimensions using a caliper twice per week. One animal inoculated with *DNM* cells was terminated earlier due to non-experimental issue and removed from the study. *DNM1* and *DNM2*: Slower and faster tumor growth groups, respectively. Each point represents the mean ± s.e. of xenografts for each group. **p < 0.01. (**B**) Distant DU145 cell clusters (H&E staining) in the kidney, lung, liver or brain of xenograft mice. Scale bar: 200 μm. (**C**) H&E and immunochemical staining for p-TGFBRI and Ki67 in xenografts. Scale bar: 200 μm. (**D**) Immunochemical staining for p-ERK in xenografts. Scale bar: 200 μm. (**E**) Traditional real-time qPCR (left panel) for cultured DU145 *TGFBRII WT* and *DNM* cells and microfluidic-based real-time qPCR for individual CTCs isolated from human DU145 xenograft mice (right panel), respectively. (**F**) Cumulative EMT gene expression in single CTCs isolated from *WT*, *DNM1* and *DNM2* hosts. ***p < 0.001; ****p < 0.0001.

Using the CRISPR/Cas9 genome-editing technique [14], we successfully altered two nucleotides in the first exon of *TGFBRII*, to increase the guide RNA efficiency, in the castration-resistant cell line DU145 (Figure 1A and Supplementary Figure S3). This genome editing could lead to putative unstable structure of the N-terminal part of TGFBRII, according to the computational prediction of its structural instability (Supplementary Figure S4). Indeed, western blot analysis confirmed the breakdown of its protein in *TGFBRII*-edited cells (Figure 1B). The disruption also prevented the TGF-β1-mediated phosphorylation of TGFBRI without changing the protein level in *TGFBRII*-edited cells relative to those of *wildtype* (*WT*) cells (Figure 1C). As a result of this dominant-negative effect, the structure of the TGFBRI/II tetrameric complex became unstable, disabling TGF-β signal propagation to SMADs (i.e., phosphorylated SMAD2/3) and to related signaling networks, including AKT and WNT (Figure 1C and 1D). Compared to *WT* cells, this *dominant-negative mutant* (*DNM*) line displayed a higher level of the epithelial marker E-cadherin (E-Cad), lower levels of mesenchymal markers N-cadherin (N-Cad) and vimentin (VM) and decreased epithelial cell adhesion molecule (EpCAM) as well (Figure 1E; see also downregulation of EMT gene markers in *DNM* cells in Figure 2E-*left*). Although there was no change in the proliferative ability, *TGFBRII*-edited cells displayed an alteration in their migratory and adhesive behaviors *in vitro*, suggesting an attenuation of TGF-β-mediated EMT in these cells (*p* < 0.01; Figure 1F). Furthermore, this deregulation of TGF-β signal transduction had a negative impact on AKT and WNT signaling, but led to a derepressed effect on ERK signaling (Figure 1C). Phosphorylation at Thr202/Tyr204 sites of p-ERK1/2 for active ERK signaling was temporarily repressed via as-yet-undefined mechanisms upon stimulation of *WT* cells (see the result of TGF-β1 stimulation at 30 min in Figure 1C). However, an increase in the phosphorylation of these sites was observed in *DNM* cells irrespective of TGF-β1 stimulation and low levels of protein in these cells. This derepression of ERK signaling was likely attributed to feedback rewiring of TGF-β transduction loops in *DNM* cells. Based on these data, we suggest that the *TGFBRII* genome-editing can disrupt the delicate balance of TGF-β-mediated oncogenic homeostasis, fortuitously activating at least one backup pathway, i.e., ERK, in *DNM* cells.

### Disrupting TGF-β signaling homeostasis leads to uncoupling of growth and metastatic potential of tumor xenografts

To determine the effect of the *TGFBRII* genome-editing on tumor growth, we inoculated *WT* or *DNM* cells into male athymic BALB/c nude mice. The majority (67%) of nine *DNM* xenografts examined (termed *DNM1*) had smaller tumor sizes relative to the *WT* group (*p* < 0.01; Figure 2A). In addition, both *DNM1* and *WT* hosts had detectable micrometastatic lesions in distant organs of their hosts (Figure 2B). Unexpectedly, we additionally observed another subset of the *DNM* group, or *DNM2*, displaying faster-growing tumors than those of *WT* and *DNM1* groups. Nevertheless, the *DNM2* subline showed no detectable metastasis in host organs. This dichotomous finding was likely attributed to the deregulation of TGF-β signaling (see attenuated p-TGFBRI staining in both DNM1/2 tumor sections in Figure 2C) that uncoupled proliferative and metastatic potential of tumor xenografts derived from the same *DNM* cell line.

Equally important is the contribution of individual host microenvironments to this uncoupling. Consistent with the *in vitro* observation, host microenvironments supported the elevation of ERK signaling activities as a feedback response in both *DNM1* and *DNM2* xenografts (Figure 2D). However, with careful examination of tumor sections we found differential staining of p-ERK in cytoplasmic and nuclear compartments of *DNM* cells. Preferential staining of nuclear p-ERK was seen in *DNM2* tumors, which could be associated with their hyperproliferative activities for faster tumor growth (see a high level of Ki67 staining in Figure 2C-*right* panels) [15]. In contrast, *DNM1* tumors displayed an increased number of cells with cytoplasmic p-ERK, known to promote cell differentiation rather than cell proliferation during tissue development [16, 17].

To determine whether cytoplasmic ERK signaling is linked to an increased metastatic potential of *DNM1* xenografts, we determined EMT expression profiles in single CTCs isolated from blood samples of corresponding hosts using a microfiltration-micromanipulator method (Supplementary Figure S5) [6]. A total of 200 CTCs isolated from *WT*, *DNM1*, and *DNM2* groups were subjected to microfluidic real-time PCR analysis. Bulk RNAs isolated from cultured *DNM* and *WT* cells were used as controls, respectively. Of 48 EMT-related and housekeeping genes analyzed, we found 10 of these loci showed differential expression patterns among CTCs isolated from these three groups of xenograft hosts (Figure 2E and Supplementary Table S1). The human origin of these CTCs was confirmed by sequencing of cDNAs of these EMT genes (data not shown). When expressed, incremental numbers and higher expression values of these 10 genes were significantly observed in CTCs isolated from *DNM1* and *WT* hosts relative to those of *DNM2* hosts, respectively (*p* < 0.0001 and *p* < 0.001, respectively; Figure 2F). Consistent with those mutant cells observed *in vitro*, CTCs of *DNM2* displayed an attenuated EMT phenotype (see Figure 1E). However, this diminished phenotype was rescued in *DNM1* CTCs, possibly leading to the maintenance of micrometastatic capability in their xenograft hosts.

### Perturbed nanomechanical properties of CTCs isolated from *DNM1* xenograft hosts

**Figure 3:**
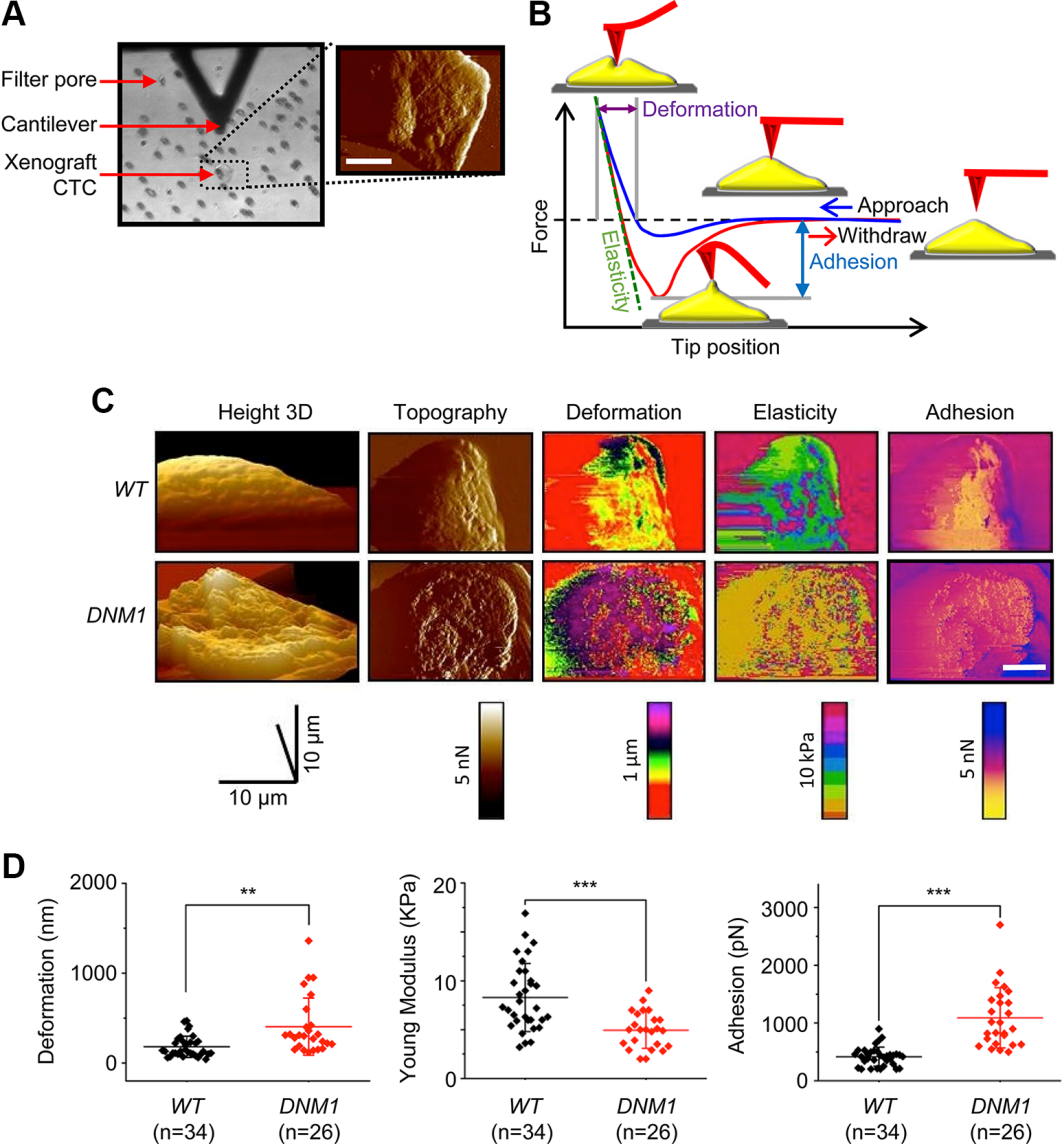
Increased deformability, elasticity and surface adhesion of CTCs isolated from *DNM1* xenograft hosts (**A**) Atomic force microscopy based measurement of nanomechanical phenotype of CTCs. The same CTC retained on a filter was identified with a light microscope (left) and then imaged with QNM AFM (right). An AFM cantilever is visible as a black triangle on the top of the image. The AFM tip is mounted perpendicularly to the cantilever and pointing toward the viewer. Filter pores are visible as regular dots. The false colored AFM scan shows the CTC imaged in a peak force error channel (relief) overlaid on the height image (topography). The color scale from black to white represents height from 0 to 10 μm. A white bar corresponds to 10 μm. (B) A force plot depicting dependence of force exercised by the tip on its distance (position) from a cell surface. A blue trace represents tip approach toward the cell and starts at the most right site of the plot, whereas the red trace illustrates a reverse direction of tip movement that begins when the tip reaches a preset value of the force. Graphical means of extracting parameters of the mechanical phenotype are also shown. (**C**) A panel of images comparing the mechanical phenotype of single *WT* and *DNM1* DU145 cells. Morphologically similar cells are included in the panel. Images are false colored. Images in the height channel are arranged as 3-D parallel projections with 45° pitch. The pitch of all other images is set to 0°. Peak force maps were used to determine cell boundaries and surface relief. In the deformation channel, leading pink and purple colors indicate a more deformable cell. Leading dark purple color observed in the elasticity channel indicates less elastic cells. An excess of yellow cell surface implies the decreased adhesion. A white scale bars corresponds to 10 μm. (**D**) Scatter plots of deformation, elasticity, and adhesion in individual CTCs isolated from blood of *WT* and *DNM1* mice. Cells isolated from the DNM1 mice were more deformable, more elastic and more adhesive (**p < 0.01; ***p < 0.001).

To determine whether CTCs isolated from blood of xenograft hosts of *DNM1* group show the enhanced potential capacity to successfully seed metastatic sites, we assessed three biophysical parameters, i.e., deformability, elasticity, and adhesion of single CTCs isolated from *WT* and *DNM1* hosts. For this purpose we employed the atomic force microscopy (AFM) based PeakForce quantitative nanomechanical (QNM) imaging [18]. In AFM, a micro-sized tip interacts with the surface of a CTC with strictly controlled force (Figure 3A). At each point of contact, the lateral and vertical tip position and the “force plot” presenting mechanical interactions of the tip with a cell surface are recorded (Figure 3B) [19–21]. The parameters extracted from the force plots are assembled into topographical and mechanical maps of a cell (Figure 3C). The parameters include elasticity describing how much pressure is needed to indent the cell in a reversible manner. The depth of maximal indentation enforced by the probe without breaking the cell membrane is a measure of reversible and non-reversible deformation. Both elasticity and deformation are crucial when CTCs are subjected to mechanical challenges during intra- and extravasation [22, 23]. The adhesion is a measure of force needed to lift the tip from the cell surface during the probe withdrawal. Cell adhesiveness is critical for assessing its invasiveness to host organs.

We found that CTCs (*n* = 26) isolated from *DNM1* hosts were almost two times more deformable than their counterpart (*n* = 34) from *WT* hosts (Figure 3D-*left*). *DNM1* CTCs appeared to be 1.3 times softer than *WT* cells (i.e., less rigid; the Young modulus as the rigidity measure is shown; Figure 3D-*middle*). This increased deformability and elasticity of *DNM1* CTCs likely facilitated their intra- and extravasation capability in xenograft hosts. Additionally, these cells were 2.5 times more adhesive than CTCs isolated from *WT* hosts (Figure 3D-*right*), highlighting their increased metastatic potential in *DNM1* hosts. This AFM approach can also be used to distinguish the aggressive nature of CTCs in prostate cancer patients (Supplementary Figure S6).

### Enumeration of CTCs and accompanying immune cells indicates high aggressiveness of *DNM1* tumors in xenograft hosts

**Figure 4:**
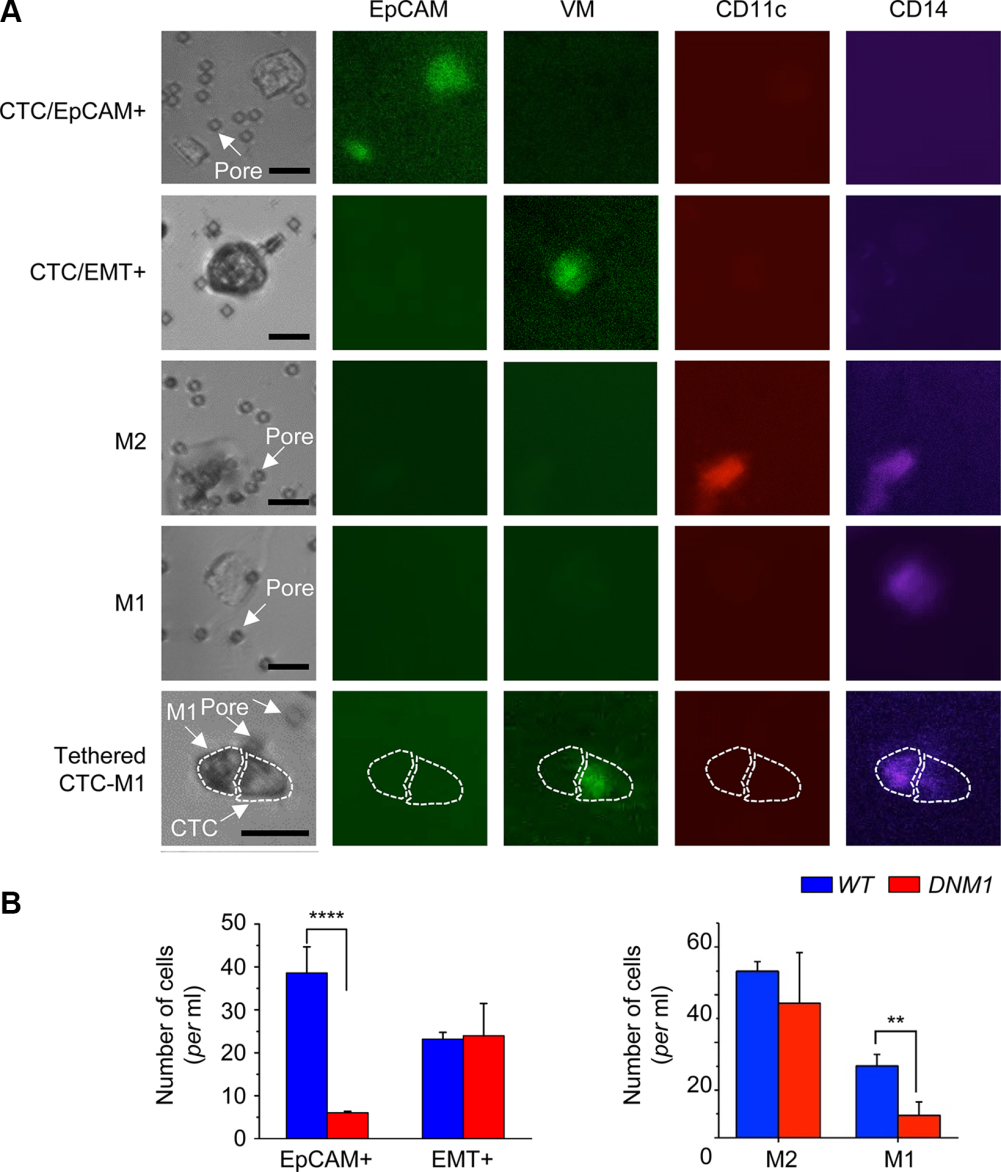
Immunocytochemical analysis of CTCs and accompanying immune cells reveals indicates high aggressiveness of *DNM1* tumors in xenograft hosts The microfiltration method of CTCs isolation retains all cells unable to pass through 6.5 μm– diameter pores when subjected to gentle pressure. (**A**) a gallery of images of cells, single or in pairs or clusters, retained on the filter and immunostained with antibodies specific for surface marker antigens, as indicated. The black bar corresponds to 25 μm. White arrows point at filter pores. The cells included CTCs, typical EpCAM^+^ or bearing EMT marker, single or in clusters. For example, the large EpCAM^+^ object in the top right corner in the first row is a cluster of at least three cells. Other large cells harvested on the filter were positive for immune cell markers and were identified as circulating macrophages. In some cases, the CTCs were found tethered to macrophages. (**B**) enumeration of distinct classes of cells revealed differences between the *WT* and *DNM1* tumor hosts. The latter had significantly less EpCAM^+^ CTCs and less M1-like (predator) macrophages than the *WT*. **p < 0.01; ****p < 0.0001.

In the microfiltration method of CTCs isolation, all cells unable to pass through the 6.5 μm-diameter pores were retained on the filter [6, 18]. Immunostaining of these cells with specific antibodies revealed their diversity. The set of cells includes not only typical CTCs, with epithelial morphology and positive for epithelial cell surface marker EpCAM, but also cells bearing EMT markers (VM), identified as CTC/EMT+. Moreover, many cells were negative for EpCAM and VM but instead positive for innate immunity cell markers (Figure 4A). These cells were usually large prompting their classification as putative circulating macrophages rather than leukocytes. We attempted to compare the immunostaining profiles of filter-retained cells in *WT* and *DNM1* xenograft hosts. Apparently, the tumors in *DNM1* xenograft hosts shed into circulation six times less of the typical EpCAM^+^ CTCs than tumors in *WT* hosts (Figure 4B-*left*). To the contrary, both *WT* and mutant hosts presented very similar numbers of CTCs negative for EpCAM but positive for EMT markers, consistent with the expression data of EMT genes presented in Figure 2F. All CTCs, both EpCAM^+^ and EpCAM^−^ VM^+^, were twice as numerous in *WT* as in *DNM1* hosts: 62 ± 5 *vs.* 30 ± 8 cells per ml of blood (*n* = 4 mice in each case), a difference that could be attributed to much larger tumors in the former (Figure 2A). Next, we analyzed the putative circulating macrophages co-purifying with CTCs. The immune cells were numerous: we found 100 ± 6 macrophages per ml of blood in *WT* hosts and 66 ± 23 cells in *DNM1* hosts (Figure 4B-*right*). Since macrophages may play very distinct physiological roles depending on their type of activation, we attempted to classify these large immune cells into pro-inflammatory M1-like and anti-inflammatory M2-like macrophages [24]. Among tumor-associated macrophages infiltrating tumor, M1 macrophages phagocytose cancer cells and are considered anti-tumor. In turn, tissue-protecting M2 macrophages are tumor-promoting [25]. We applied the classification into circulating macrophages harvested by microfiltration and noted striking differences between the *WT* and *DNM1* xenograft hosts. While the number of M2-like macrophages was similar in both types of xenograft hosts, the number of M1-like, presumably anti-tumor macrophages was more than three times lower in mutant than in the *WT* (*p* < 0.01; Figure 4B-*right*)

Most of the on-filter analyzed CTCs were single but some of them were grouped in clusters with other CTCs or were tethered to macrophages. Clustered CTCs are considered to be more aggressive than single CTCs [26]. On the other hand, the presence of CTC-macrophage pairs suggests interactions between these cells, for example M1 macrophage destroying CTC or M2 macrophage protecting CTC. The number of detected clusters and pairs was limited, less than 10% of all counted cells: on average 5 ± 4 per ml of blood in *WT* (11 cases analyzed) and 7 ± 4, in *DNM1* (15 cases analyzed). Such low count did not support statistical analysis; however there were notable trends in partition of distinct types of pairs and clusters. In general, we found more CTC clusters in *DMN1* than in *WT* hosts: 2.7 *vs.* 1.7 cells/ml of blood samples, respectively, suggesting high aggressiveness of tumor-shed cells in the former. We observed more clusters of EMT^+^CTCs than clusters of EpCAM^+^CTCs, in *WT* and especially in *DNM1* hosts (WT: 1.2 *vs.* 0.5, *DNM1*: 2 *vs.* 0.7 cells/ml of blood, respectively). Interestingly, we found more pairs of M2-like macrophages with CTCs than M1-like macrophages (WT: 2.9 *vs.* 0.7 cells/ml of blood), and the difference was even more pronounced in *DNM1* hosts (3.2 *vs*. 0.6 cells/ml of blood). Based on these observations, we speculate that the *DNM1* phenotype is a result of not only properties of xenografted cells alone, but also an organismal response to the tumor, shaped by the xenografted cells' properties.

Enumeration of not only EpCAM^+^CTCs but also EMT^+^CTCs, circulating macrophages and pairs/clusters of cells may be a viable option for characterization of tumor aggressiveness in human prostate cancer patients. As shown in Supplementary Figure S7A, the distinct types of CTCs, macrophages and pairs/clusters were detectable in samples of filtrated blood from metastatic prostate cancer patients. The diversity of partitions of cell types is apparent in Supplementary Figure S7B, with enumeration performed for three randomly chosen patients.

### Combination of *TGFBRII*-disruption and ERK inhibition attenuates aggressive potential of *TGFBRII*-edited prostate cancer cells

**Figure 5:**
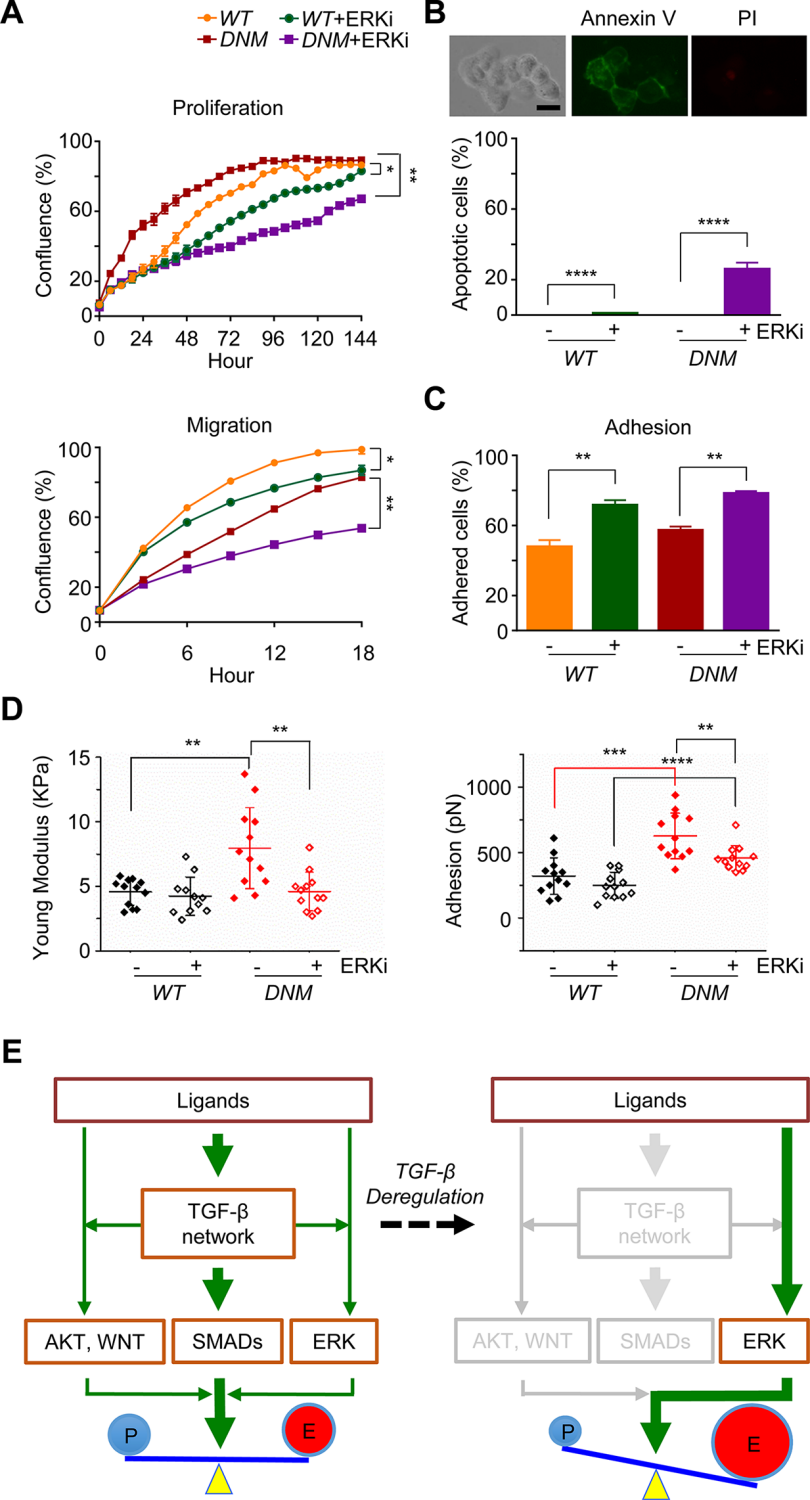
Combination of *TGFBRII*-disruption and ERK inhibition attenuates aggressive potential of *TGFBRII*-edited prostate cancer cells (**A**) Cell proliferation and migration (wound healing), respectively, using IncuCyte^®^ ZOOM live-cell kinetic imaging system. DU145 *TGFBRII WT* and *DNM* cells were treated with 10 μM of ERK inhibitor SCH772984 (ERKi) or vehicle alone. *p < 0.05; **p < 0.01. (**B**) Cell apoptosis/death analysis. DU145 *TGFBRII WT* and *DNM* cells treated with or without ERKi (10 μM) for 24 hours and stained with FITC annexin V and propidium iodide. Annexin V positive cells were counted against total cells. Scale bar: 50 μm. ****p < 0.0001. (**C**) Cell adhesion assay using IncuCyte^®^ ZOOM live-cell kinetic imaging system. DU145 TGFBRII *WT* and *DNM* cells were treated with 10 μM of ERK inhibitor SCH772984 (ERKi) or vehicle alone. **p < 0.01. (**D**) AFM analysis of cultured DU145 TGFBRII *WT* and *DNM* cells treated with or without 3 μM of ERKi for 24 hours. **p < 0.01; ***p < 0.001; ****p < 0.0001. (**E**) A proposed model of feedback rewiring of deregulated TGF-β signaling sustains EMT of CTCs. In *TGFBRII*-disrupted prostate cancer cells, both SMADs-dependent and -independent pathways were suppressed with the exception of positive feedback of ERK signaling, resulting uncoupled cell growth and metastasis capability.

It's well known that tumor-suppressor functions of TGF-β signaling are selectively shut down while exerting its pro-tumoral function in advanced prostate cancer [12]. To determine the potential benefit of inhibiting ERK activity along with *TGFBRII*-disruption in cultured *DNM* cells, we treated *WT* and *DNM* cells with SCH772984, an ATP-competitive ERK1/2 inhibitor (ERKi), at the dosage of 10 μm according to previous report [27]. As expected, 10 μM of ERKi incubation significantly reduced cell proliferation and migration in *WT* cells (*p* < 0.05; Figure 5A); in *DNM* cells, ERKi at the same dosage also reduced cell growth and the inhibitory effect was much stronger than that in *WT* cells (*p* < 0.01; Figure 5A-*top*). Cell apoptosis assay revealed that 10 μM of ERKi treatment induced apoptosis in *DNM* cells (Figure 5B). Therefore, both *WT* and *DNM* cells had similar growth curve without ERKi incubation since elevated p-ERK1/2 in *DNM* cells could compensate the expected inhibitory effects of disruption of *TGFBRII* gene on TGF-β signaling pathways (Figure 1F), but when cells were treated with ERKi, cell apoptosis was apparently involved especially in *DNM* cells. Moreover, 10 μM of ERKi significantly suppressed cell migration of cultured *DNM* and its effect was much more robust than that of *WT* cells (*p* < 0.01; Figure 5A-*bottom*). Cell adhesion assay revealed that both *WT* and *DNM* cells were more adhesive when incubated with 10 μM of ERKi for a short time period (0.5 hour; Figure 5C); however, long time period of treatment with ERKi at either 10 μM (data not shown) or lower dosage of 3 μM (*p* < 0.05; Figure 5D-*right*) reduced cell adhesion in *DNM* cells as analyzed by AFM. AFM analysis further showed an increase in cell stiffness in *DNM* cells induced by ERKi treatment at both 10 μM (data not shown) and 3 μM (*p* < 0.05; Figure 5D-*left*) while no significant difference was observed in *WT* cells. Together, inhibiting ERK signaling in *DNM* cells could reduce their aggressive natures. These findings support the existence of positive feedback of ERK pathway in *TGFBRII*-edited *DNM* cells and provide a novel combined targeting of both TGF-β and ERK signaling pathways in advance prostate cancer.

## DISCUSSION

Cancer growth and metastasis is a highly complex process that requires fine-tuning of its proliferative and migratory potential. In the present study, we demonstrate that TGF-β signaling network plays such a role of regulating both growth and metastasis in mCRPC cells. The network actions are well-orchestrated, depending on the crosstalk between TGF-β and other signaling collaborators in cancer cells. Nevertheless, this TGF-β-mediated network can be deregulated through a dominant-negative effect *via* genome-editing of its cognate receptor *TGFBRII*. In particular, we observed downregulation of at least two signaling collaborators (i.e., AKT and WNT) and derepression of ERK signaling. This derepression is likely triggered by a feedback mechanism when the main network traffic is being blocked in *TGFBRII*-edited cells (see the proposed model in Figure 5E). Although other feedback pathways may be present and remain to be elucidated, we suggest that derepression of ERK signaling greatly perturbs oncogenic homeostasis, leading to uncoupling of growth and metastasis in *DNM* tumor xenografts. Specifically, we observed nuclear p-ERK predominantly present in a subset of highly proliferative *DNM* tumors (*DNM2*). Nuclear p-ERK is known to interact with transcription factors MYC, ETS, and CREB for transcription upregulation of genes involved in anti-apoptosis and proliferative functions [17, 28]. On the contrary, the majority of *DNM* tumors (*DNM1*) displayed nuclear-to-cytoplasmic shuttling of p-ERK, which phosphorylates SLUG for EMT initiation and other transcription factors for cell motility and invasion [8, 9]. This p-ERK switching may compensate the loss of EMT functions originally engendered by TGF-β signaling. From this study, it is also clear that host microenvironments play a critical role in directing the nuclear-to-cytoplasmic shuttling of p-ERK that switches cancer cells from a state of proliferation to a state of migration and invasion, and *vice versa*. Therefore, molecular analysis of host stroma surrounding *DNM* tumors can be considered in a future study to identify different external stimuli that trigger the growth-and-metastasis uncoupling of a tumor through p-ERK switching.

The present finding has an important therapeutic implication when anti-TGF-β therapies are used as single agents for treating advanced cancer patients, including mCRPC. Targeting TGF-β signaling has been an emerging therapeutic strategy due to its roles in the development of cancer, including TGF-β-driven EMT and metastasis [12, 29, 30]. In addition to TGF-β, one caveat is that other growth factors can initiate EMT through receptor tyrosine kinases and other signaling pathways, including WNT, Notch and Hedgehog [8]. Indeed, the present study revealed the feedback rewiring in advanced prostate cancer cells when TGF-β signal transduction is blocked, and activation of p-ERK appears to be a backup pathway to compensate the loss of EMT in *TGFBRII*-edited cells. As a result, EMT is being restored with a subsequent increase in the migratory capability of these cancer cells in xenograft hosts, resulting in distant micrometastasis. Our study demonstrated that additional treatment with ERK inhibitor SCH772984 could suppress cell growth and induce apoptosis of *DNM* cells. Furthermore, *in vivo* study demonstrated dichotomous tumor growth patterns in mice inoculated with *DNM* cells when compared to *WT* group, and both p-ERK1/2 and Ki67 staining supported the correlated relationship of p-ERK and cell proliferation and tumor growth. We expected that inhibition of ERK signaling pathway would reduce tumor growth in *DNM2* mice, and based on this, we plan to explore a combined strategy (i.e., ERK + TGF-β inhibitors) for treating mCRPC in animal models. This future study has an important therapeutic implication in that dual blockade of the key and feedback pathways for EMT is an additional option for treating advanced prostate cancer patients.

To monitor treatment efficacy of these inhibitors, CTCs can be considered as “liquid biopsy” for longitudinal evaluation of mCRPC patients. We have demonstrated that nanomechanical characters of CTCs and enumeration of CTCs and host macrophages provide critical information regarding decreased or increased metastatic potential of these circulating cells during the development of prostate cancer. Since metastatic tumors are often inaccessible, CTCs can be isolated and repopulated into organoids for establishing patient-derived xenografts. Similar to the concept of mouse hospital [31], these CTC-derived xenografts (CDXs) are first used to test the efficacy of combined therapies [32]. Single CTCs are then isolated from CDX hosts to characterize their nanomechanical properties using AFM and to monitor their interactions with macrophages in the bloodstream. Additionally, through single-cell expression profiles of EMT genes, we may identify responder and non-responder signatures of the combined TGF-β+ERK treatment or others. This parallel preclinical testing provides valuable information of combination optimization for treating mCRPC.

It is well accepted that mechanisms independent of canonical androgen receptor (AR) signaling maintain AR activity in CRPC. Therefore, the use of advanced prostate cancer DU145 cell line that lacks functional AR signaling can provide additional avenues for effective management of CRPC. Nevertheless, our current proof of principle study warrants further investigations using patient-derived xenografts (PDXs) and/or CDXs in co-clinical trials [31].

In summary, CRISPR/Cas9 genome-edited *TGFBRII* leads to the blocking of TGF-β signaling pathways in advanced prostate cancer cells, triggering positive feedback activation of ERK that uncouples malignant cell growth and metastasis capability in a xenograft mouse model. This uncoupling may adversely enhance EMT-mediated metastasis through this backup pathway. Therefore, combined targeting of TGF-β and its backup partner ERK can be an attractive strategy to block EMT in mCRPC patients.

## MATERIALS AND METHODS

### Cell culture

Human prostate cancer cell line DU145 (ATCC HTB-81) and CRISPR/Cas9-mediated *TGFBRII*-edited, *via* lenti TGFBRII-gRNA/Cas9, DU145 cells (Supplementary Methods and Figure S1) were grown in RPMI 1604 supplemented with 10% fetal bovine serum and 1X penicillin/streptomycin (all from Life Technologies, Grand Island, NY) in a 100% humidified 5% CO_2_ incubator at 37°C.

### RNA isolation and real-time quantitative PCR

Cellular total RNA were extracted from *WT* and lenti TGFBRII-gRNA/Cas9-treated cells using Quick-RNA MiniPrep Kit (ZYMO Research, Irvine, CA), respectively. One μg of RNA were subjected to reverse transcription using SuperScript^®^ VILO^™^ MasterMix (Invitrogen Corporation), followed by qPCR, using iTaq^®^ Universal SYBR Green Supermix (Bio-Rad Laboratories, Inc., Hercules, CA) on Applied Biosystem^®^ 7900HT Fast Real-Time PCR System (Life Technologies), for primers: BMP-7, CD133, EpCAM, FOXC2, FZD4, GSK3B, MMP9, SNAIL2, SOX9, TCF3, TGF-β2, TGF- β3, TWIST1, WNT5A, WNT5B, ZEB1, ZEB2, and GAPDH (all from Sigma-Aldrich Co. LLC.; Supplementary Table S1) [6].

### Western blot analysis

Cellular total proteins were extracted from DU145 *WT* or lenti TGFBRII-gRNA/Cas9-treated cells, treated with or without 5 ng/ml of recombined human TGF-β1 (R&D Systems, Minneapolis, MN) for different duration. Fifty μg of proteins were subjected to SDS-PAGE, blotting and immunoreaction with antibodies: anti-TGFBRII (full length, FL; Santa Cruz Biotechnology, Inc., Santa Cruz, CA), anti-TGFBRII (N-terminal, NT; EMD Millipore Corporation, Billerica, MA), anti-TGFBRI (Cell Signaling Technology, Danvers, MA), anti-phosphorylated-TGFBRI (p-TGFBRI; Abcam, P.L.C., Cambridge, MA), anti-SMAD2/3 (Cell Signaling Technology), anti-phosphorylated-SMAD2 (p-SMAD2; Cell Signaling Technology), anti-phosphorylated-SMAD3 (p-SMAD3; Cell Signaling Technology), anti-SMAD4 (Cell Signaling Technology), anti-phosphorylated-SMAD4 (p-SMAD24; Cell Signaling Technology), anti-PERK1/2 (Cell Signaling Technology), anti-phosphorylated-ERK1/2 (p-ERK1/2; Cell Signaling Technology), anti-AKT (Cell Signaling Technology), anti-phosphorylated-AKT (p-AKT; Cell Signaling Technology), anti-N-cadherin (N-Cad; BD Biosciences, San Jose, CA), anti-E-cadherin (E-Cad; Cell Signaling Technology), anti-fibronectin (FN; BD Biosciences), anti-vimentin (VM; Cell Signaling Technology), anti-epithelial cell adhesion molecule (EpCAM; Stemcell Technologies, Vancouver, British Columbia, Canada), anti-SNAIL (Cell Signaling Technology) and α-tubulin (Santa Cruz Biotechnology, Inc.), respectively. After incubation with secondary antibody, proteins on polyvinylidene difluoride (PVDF) membranes were incubated with Clarity™ Western ECL Blotting Substrate (Bio-Rad), followed by X-film exposure and development.

### Cell proliferation, adhesion and migration assays

Cells were seeded into 96-well tissue culture plate (Invitrogen Corporation) at the density of 2,000–10,000/ml. Cell growth was monitored by measuring confluence for 72–144 hours with IncuCyte ZOOM live-cell kinetic imaging system (Essen BioScience, Ann Arbor, MI). Cells were seeded into 96-well tissue culture plate at the density of 2,000–10,000/well. Thirty minutes after seeding, the plate was washed twice with 1X Dulbecco's Phosphate-Buffered Saline (DPBS; Life Technologies) and replaced with fresh complete medium. Initial images after seeding and images after medium replace were captured with the IncuCyte ZOOM live-cell kinetic imaging system. The percentages of the population of adhered cells were calculated by measuring confluence and compared between DU145 *WT* and lenti TGFBRII-gRNA/Cas9-treated cells. Cells were seeded into Image Locker 96-well tissue culture plate (Essen BioScience) at the density of 50,000/ml. Three hours later, scratch wound area was created by using Essen 96-well WoundMaker (Essen BioScience). Wound healing (cell migration) was monitored for 18 hours and images were captured with the IncuCyte ZOOM live-cell kinetic imaging system every 2 or 3 hours.

### Cell death/apoptosis assay

Cells were treated with ERK inhibitor (ERKi or SCH772984; Selleck Chemicals, Houston, TX) for 24 hours, and apoptosis was determined using FITC Annexin V/Dead Cell Apoptosis Kit (Invitrogen Corporation) per manufacturer's instructions. Briefly, cells grown on chamber slides were washed with cold 1X PBS and 1X annexin V-binding buffer, incubated in FITC annexin V and propidium iodide (PI) for 15 minutes, washed with 1X annexin V-binding buffer again and microscopic pictures were taken with an inverted Evos *fl* digital fluorescence microscope (Advanced Microscope Group or AMG, Bothell, WA).

### Xenograft mouse model

Twenty 4-week-old male athymic nude mice were purchased from Envigo (Indianapolis, IN). The animals were housed under pathogen free conditions. The institutional animal use committee approved all animal experiments performed in this study. Exponentially growing human prostate cancer DU145 *WT* and lenti TGFBRII-gRNA/Cas9-treated cells were subcutaneously inoculated in the flank of 5-week-old mice at 1.5 × 10^6^ cells per inoculum. Xenografts were measured externally in two dimensions twice per week using a caliper. Xenograft volume (V; mm^3^) was determined by the following equation: V = (L × L × W)/2, where L is the length and W is the width of a xenograft. Data were presented as mean ± s.e.m. Blood samples were collected for CTCs isolation (see the below for details). Tumor tissues and other tissues of liver, lung, spleen, kidney, bone and brain were fixed in 10% neutral buffered formalin overnight, except 48 hours for brain tissues, at room temperature, embedded in paraffin and sectioned, followed by routine H&E staining and (tumor tissues) immunochemistry (IHC). For IHC, deparaffinized sections were pre-treated with 3% hydrogen peroxide and normal goat serum, respectively, followed by incubation in PBS with antibodies against TGFBRII, TGFBRI, p-TGFBRI, ERK1/2, p-ERK1/2, AKT, p-AKT, and anti-Ki67 (Invitrogen Corporation), respectively, overnight at 4°C, and biotinylated-secondary antibodies subsequently for 1 hour at room temperature, and then incubated in AB Complex (Vectorstain^®^ Elite ABC Kit; Vector laboratories, Inc., Burlingame, CA) at room temperature for 45 minutes prior to 3,3′-diaminobenzidine (DAB) staining (ImmPACT™ DAB Peroxidase Substrate; Vector Laboratories, Inc.) prior to dehydration and mounting.

### Isolation of circulating tumor cells from xenograft hosts

Mice inoculated with DU145 *TGFBRII WT* or lenti TGFBRII-gRNA/Cas9-treated cells were anesthetized by isoflurane inhalation, 0.5–1.0 ml of blood from each mouse was collected via cardiac puncture and transferred into an ethylenediaminetetraacetic acid (EDTA)-treated K2 EDTA tube for CTCs isolation per instructions of *ScreenCell*^®^ CC filtration kit (ScreenCell, Westford, MA) as previously reported [6]. Briefly, blood samples were homogenized in *ScreenCell*^®^ LC dilution buffer, filtered through *ScreenCell*^®^ filtration module, and then cells retained on the filter were dissociated and stained for CD45 (PE-conjugated antibody) and EpCAM (FITC conjugated antibody), followed by single cell isolation, using a Narishige micromanipulator and Ferty Syringe Plus Microinjector (Origio MidAtlantic Devices, Mount Laurel, NJ) with the inverted Evos *fl* digital fluorescence microscope, collected into 4.5 μl lysis buffer (Invitrogen Corporation) individually.

### Single-cell gene expression profiling

Single cell lysis underwent through reverse transcription-specific target amplification (RT-STA; preamplification), followed by microfluidic-based real-time qPCR on BioMark HB MX/HX system (Fluidigm Corporation, South San Francisco, CA) with 1X SsoFast Eva-Green supermix with low ROX (Bio-Rad) and 1X DNA binding dye sample loading reagent (Fluidigm Corporation) following the protocol of single-cell gene expression (Fluidigm Corporation) as previously reported [6]. PCR primers of selected genes for expression profiling were selected from the PrimerBank database (Supplementary Table S1) [6]. In each chip assay, universal RNA (200 pg) from human normal tissues (BioChain Institute, Inc., Newark, CA) and no template control (NTC) served as positive and negative controls, respectively. We also subjected proportion of preamplification products of individual CTCs to sequencing to verify human originality of isolated CTCs and rule out the possibility of mouse cell contamination.

### Atomic force microscopy analysis of xenograft CTCs

CTCs preparation followed the above CTCs isolation procedure, except that cells were kept on filter in DMEM supplemented with 5% fetal bovine serum and subjected to immediate AFM analysis after the staining as we previously reported [6, 18]. Briefly, cells were retained on the filter and the filter was mounted on a 60 mm tissue culture dish, without any additional processing or immobilization, cells were imaged using a Nanoscope Catalyst AFM (Bruker, Billerica, MA), with SCANASYST-AIR (Bruker) probes, mounted on a Nikon Ti inverted epifluorescent microscope. The nanomechanical phenotype data for each individual cell were captured, and cell elasticity, deformation and adhesion were analyzed with Nanoscope Analysis software v.4.1. The measurements of the elastic modulus followed the rules published by Sokolov assuming a high heterogeneity of cell surface properties (brush and rigidity); calculations were performed based on the Sneddon model that approximates the mechanics of conical tip interactions with an object. Elasticity is expressed in units of pressure (Pascals, Pa) as the Young's modulus. Higher values of the Young's modulus correspond to more rigid (less elastic) objects. Deformation is presented in units of length and assesses the depth of a cell indentation at a selected point by a preset force. Deformation includes elastic (reversible) and non-elastic (nonreversible; plastic) components, without cell fracturing. Adhesion is measured in units of force (Newtons, N) and quantifies capability of a cell to cling to another material.

### Immunofluorescence analysis of xenograft CTCs and macrophages

CTCs preparation followed the above CTCs isolation procedure, except that cells retained on the filter were not dissociated and they were stained against CD11c, CD14 and VM in addition to EpCAM and CD45. We counted the numbers of M1-like (CD11c^−^) or M2-like (CD11c^+^) macrophages, single or tethered to CTCs, and distinguished EMT-CTCs (VM^+^, EpCAM^+^ or EpCAM^−^) from non-EMT-CTCs (EpCAM^+^ VM^−^) as well.

### Statistical analysis

Student two-tail *t* test and one-way ANOVA were performed using GraphPad Prism software version 6.0.5, and *p* < 0.05 was considered statistically significant. Results are expressed as the mean ± s.d. unless indicated otherwise.

## SUPPLEMENTARY MATERIALS FIGURES AND TABLE




