# Supplementary material for: TGF-β signal rewiring sustains epithelial-mesenchymal transition of circulating tumor cells in prostate cancer xenograft hosts
Source: Oncotarget. 2016 Oct 21;7(47):77124–37. doi: 10.18632/oncotarget.12808 (PMC5363574; doi:10.18632/oncotarget.12808)
Supplement: Supplementary file 1 [file oncotarget-07-77124-s001.pdf]

TGF-β signal rewiring sustains epithelial-mesenchymal transition of circulating tumor cells in prostate cancer xenograft hosts

Supplementary Materials

Cell culture

Human embryonic kidney 293T cells (ATCC CRL-3216), and DU145 cells, were grown in RPMI 1604 supplemented with 10% fetal bovine serum and 1X penicillin/streptomycin (all from Life Technologies, Grand Island, NY) in a 100% humidified 5% CO2 incubator at 37°C.

Lenti TGFBRII-gRNA/Cas9 constructs, 293T transfection, and transduction of DU145

The targeting of single guide RNA (gRNA), designed to target N-terminal of human *TGFBRII* gene to increase the gRNA efficiency, using Michael Boutros lab's Target Finder (E-CRISP; http://www.e-crisp.org/E-CRISP/designcrispr.html), was introduced into a lentiviral vector lentiCRISPR, a gift from Dr. Feng Zhang (Addgene plasmid # 49535; Cambridge, MA), delivering Cas9, a gRNA and a puromycin selection marker into target cells. 293T cells, seeded in 100mm tissue culture dishes (Invitrogen Corporation, Carlsbad, CA) were transfected with 4 µg each of lenti TGFBRII-gRNA/Cas9, psPAX2 and pMD2.G (both are gifts from Didier Trono; Addgene plasmid # 12260 and # 12259, respectively), using Lipofectamine 2000 (Invitrogen Corporation), including controls without plasmid and/or Lipofectamine 2000, per the manufacturer's instructions. Fresh medium was replaced the following day and, after an additional 24 hours, the supernatant was harvested by centrifugation and filtered through 0.45-µm filters.

DU145 cells in 6-well tissue culture plates (Invitrogen Corporation) were infected with the filtered supernatant of 293T, together with 8 µg/ml of polybrene (Sigma-Aldrich Co. LLC., St Louis, MO), followed by medium renewal supplemented with 0.5 µg/ml of puromycin (Life Technologies) the following day, and then after an additional 24 hours, cells were expanded and seeded into 100mm tissue culture dishes (Invitrogen Corporation). Controls included the supernatant of 293T without plasmid and/or Lipofectamine 2000. About 2 weeks later, single cell clones were picked up individually and placed into 24-well tissue culture plates (Invitrogen Corporation), and later into 6-well tissue culture plates (Invitrogen Corporation). Genomic DNA (gDNA) was extracted from cells, subjected to PCR amplification using PyroMark PCR kit (QIAGEN, Valencia, CA), and subsequently purified products were sequenced to confirm gene editing.

Validation of the prognostic effect of TGFBRII mRNA in prostate cancer dataset

We used an online biomarker validation tool and database SurvExpress (http://bioinformatica.mty.itesm.mx:8080/Biomatec/SurvivaX.jsp) [1] to perform survival analysis, providing Kaplan-Meier log rank analysis and risk assessment using *TGFBRII* gene list as input to a Cox proportional-hazards regression. The largest normalized dataset (GSE10645-GPL5858) with 596 samples that included overall survival times and survival after radical retropubic prostatectomy (Survival_After_RRP) was used. For replicated genes, we selected the average expressed probe and maximize risk groups. Overall survival times, relapse-free survival and risk analysis were performed in which a predicted risk for a prostate cancer patient genetic profile was determined. The subjects were then partitioned into low risk and high risk groups [1].

Computational prediction of the stability of mutation TGFBRII

We used I-Mutant2.0 (http://folding.biofold.org/i-mutant/i-mutant2.0.html) [2] to predict the potential stability change of TGFBRII upon amino acid substitution (T23I), using protein structure as input without changing any default parameters.

Prostate cancer patient blood samples and CTCs analysis

Eight ml of blood samples from PrCa patients were used to isolate CTCs for AFM and/or immunofluorescence analyses as reported previously [3]. We calculated nanomechanical parameters including elasticity, cell deformation and adhesion, as described [3]. Following staining of the same cells with specific fluorescently labeled antibodies we counted EMT- and non-EMT-CTCs as well as large innate immunity cells identified as macrophages, copurifying with CTCs. We identified as M1-like or intermediate cells with high levels of CD14 and/or CD80 antigens, and as M2-like cells with low or

no immunofluorescence-detectable expression of CD14 or CD80, but positive for CD11c and/or CD45. The institutional review board approved all clinical research experiments in this study.

REFERENCES

1. Aguirre-Gamboa R, Gomez-Rueda H, Martinez-Ledesma E, Martinez-Torteya A, Chacolla-Huaringa R, Rodriguez-Barrientos A, Tamez-Pena JG, Trevino V. SurvExpress: an online biomarker validation tool and database for cancer gene expression data using survival analysis. PLoS One. 2013; 8:e74250. doi: 10.1371/journal.pone.0074250.

2. Capriotti E, Fariselli P, Casadio R. I-Mutant2.0: predicting stability changes upon mutation from the protein sequence or structure. Nucleic Acids Res. 2005; 33:W306-10. doi: 10.1093/nar/gki375.

3. Osmulski P, Mahalingam D, Gaczynska ME, Liu J, Huang S, Horning AM, Wang CM, Thompson IM, Huang TH, Chen CL. Nanomechanical biomarkers of single circulating tumor cells for detection of castration resistant prostate cancer. Prostate. 2014; 74:1297-307. doi: 10.1002/pros.22846.

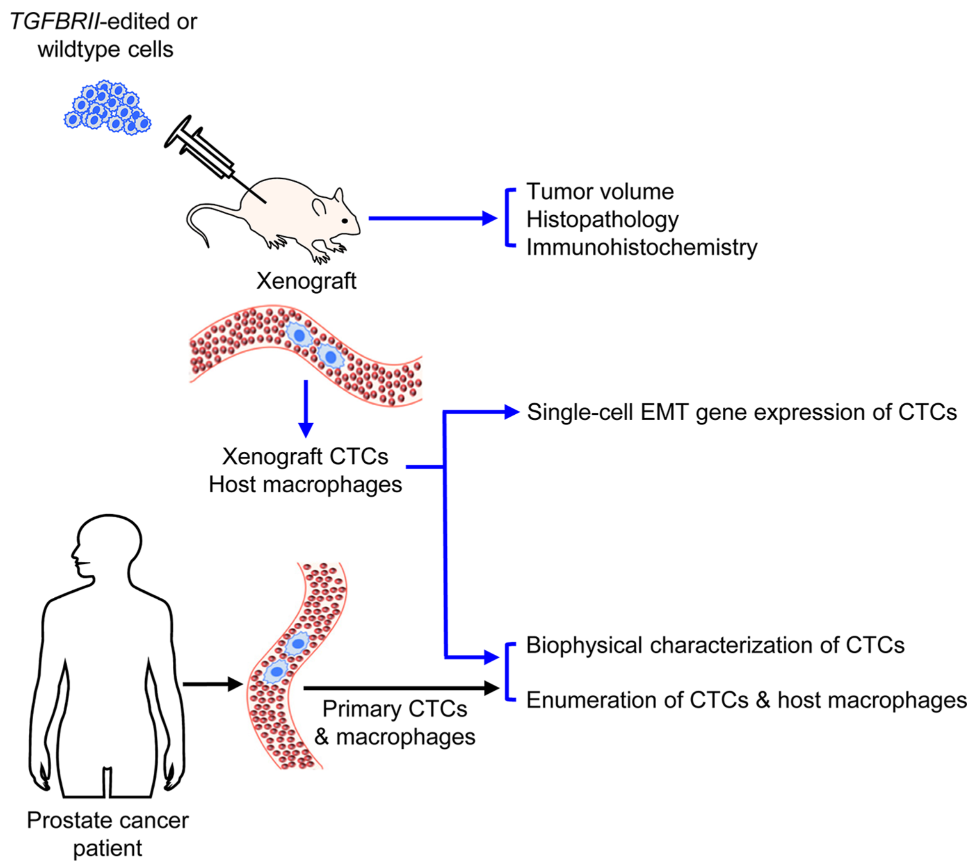

Supplementary Figure S1: Scheme of the experimental design. DU145 wildtype (*WT*) and *TGFBRII*-edited cells were injected in nude mice, respectively. To determine the histopathological changes in xenograft hosts we also isolated single CTCs from blood samples of xenografted mice or PrCa patients and subjected to (i) single cell expression of EMT-related genes, or (ii) nanomechanical phenotype analysis using atomic force microscopy and (iii) immunocytochemical analysis and enumeration of distinct types of CTCs and co-purifying macrophages.

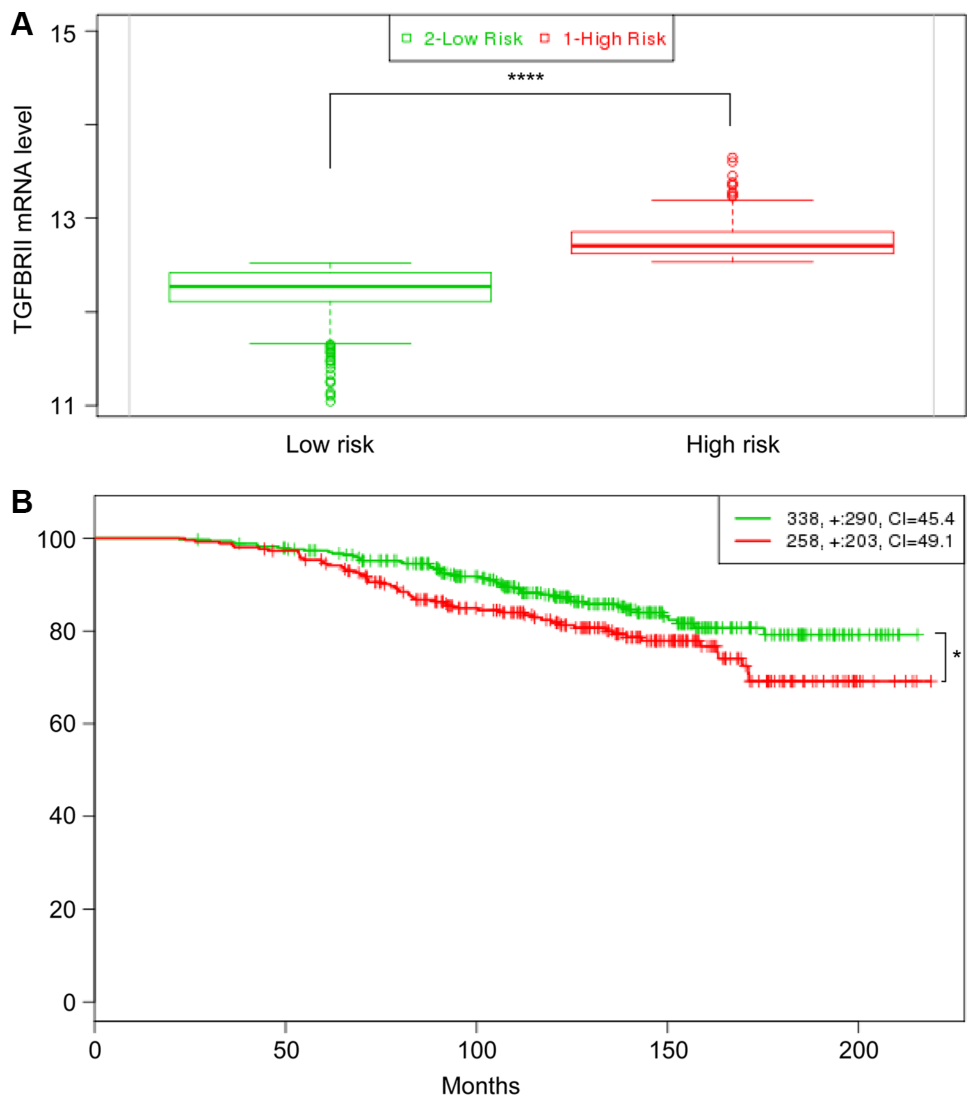

Supplementary Figure S2: Analysis of *TGFBRII* gene expression and survival after radical prostatectomy in patients with prostate cancer. (**A**) Box plots generated by the SurvExpress program show the mRNA expression levels of *TGFBRII* and the *p*-value associated with Student's *t*-test of the difference. ****$p < 0.0001$. (**B**) Kaplan-Meier survival curves constructed using the SurvExpress program for the analysis of patient samples of prostate cancer from Kollmeyer-Jenkins prostate GSE10645-GPL5858 dataset. Green and red indicate low and high expression groups, respectively. The insets in the top-right show the number of individuals, the number censored, and the Concordance Index (CI) of each risk group. Markers (+) represent censoring samples. *$p < 0.05$.

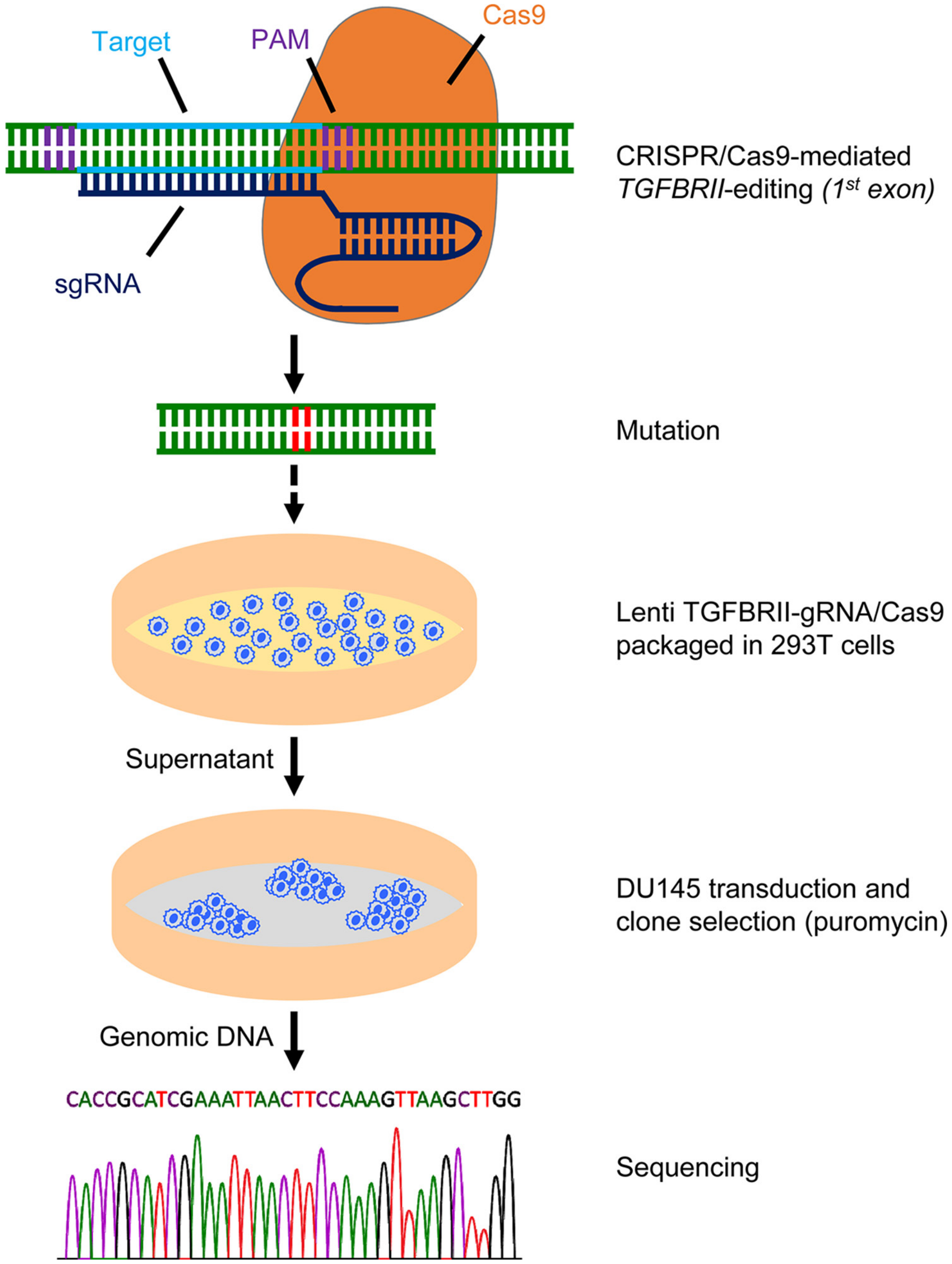

Supplementary Figure S3: CRISPR/Cas9-mediated *TGFBRII*-edition in DU145 cells. Lenti TGFBRII-gRNA/Cas9 was constructed to edit first exon of *TGFBRII*, and packaged in 293T cells. DU145 cells were then infected with lenti TGFBRII-gRNA/Cas9, harvested from the supernatant of 293T cells, and single colonies were expanded for further culture. Genomic DNA was extracted and subjected to sequencing to confirm *TGFBRII*-editing.

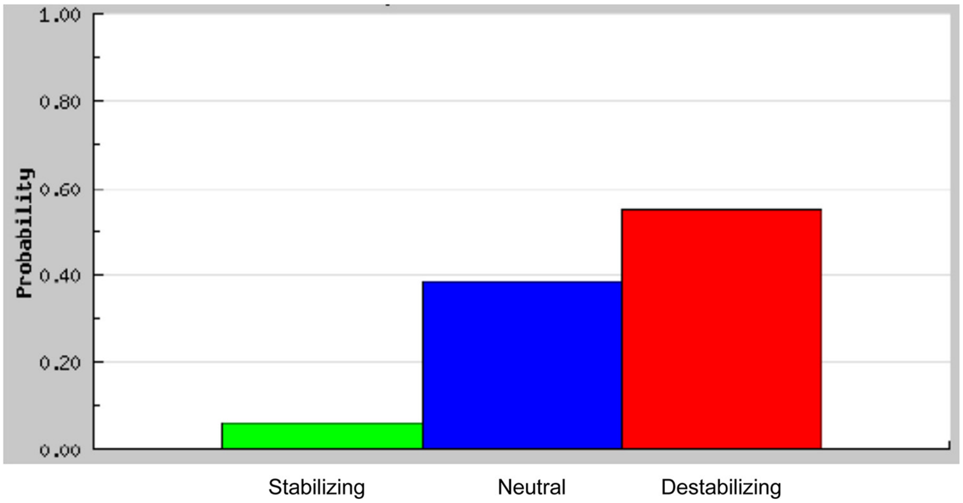

Supplementary Figure S4: Computational prediction of stability of mutant TGFBRII protein. An online tool I-Mutant2.0 was used to predict the potential stability change of TGFBRII upon amino acid substitution (T23I), using protein structure as input with default parameters.

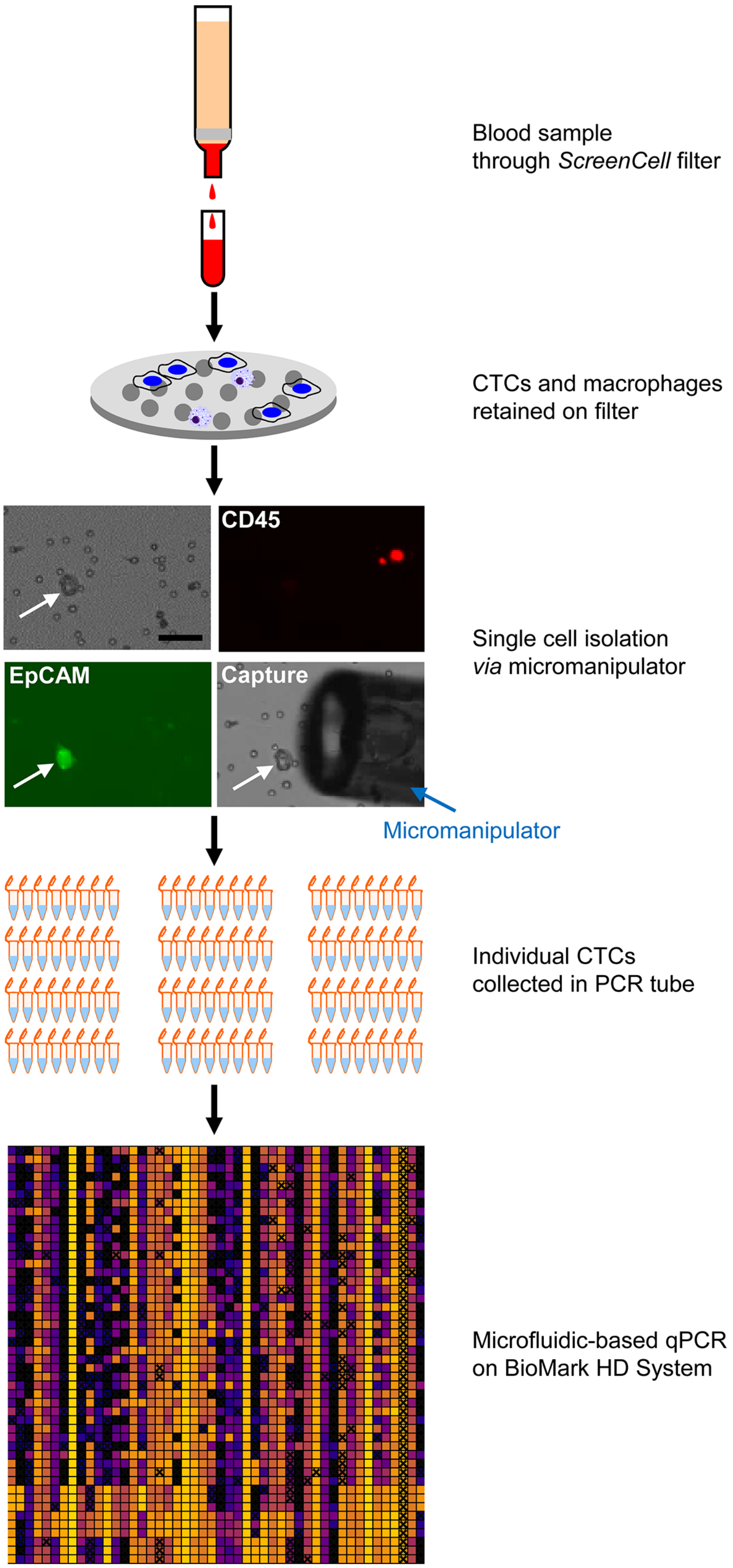

Supplementary Figure S5: Scheme of single CTCs isolation and gene profiling analysis. Individual blood sample from xenograft hosts was collected to isolate single CTCs using *ScreenCell*® CC filtration kit along with immunofluorescence staining with CD45 and EpCAM. , Filtration was followed by single cell isolation, using a micromanipulator with a fluorescence microscope; subsequently cells were individually collected into lysis buffer, and subjected to microfluidic-based real-time qPCR. Scale bar: 50 µm.

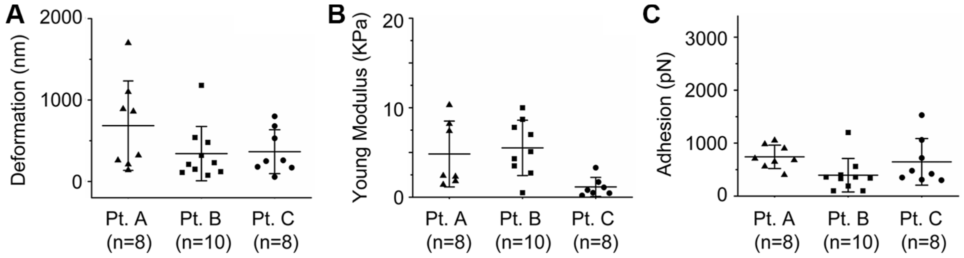

Supplementary Figure S6: AFM analysis of single CTCs isolated from blood samples of patients with prostate cancer. Scatter plots of deformation (**A**), elasticity (**B**), and adhesion (**C**) in individual CTCs isolated from blood of 3 individual prostate cancer patients showing the mechanical diversity of the CTCs.

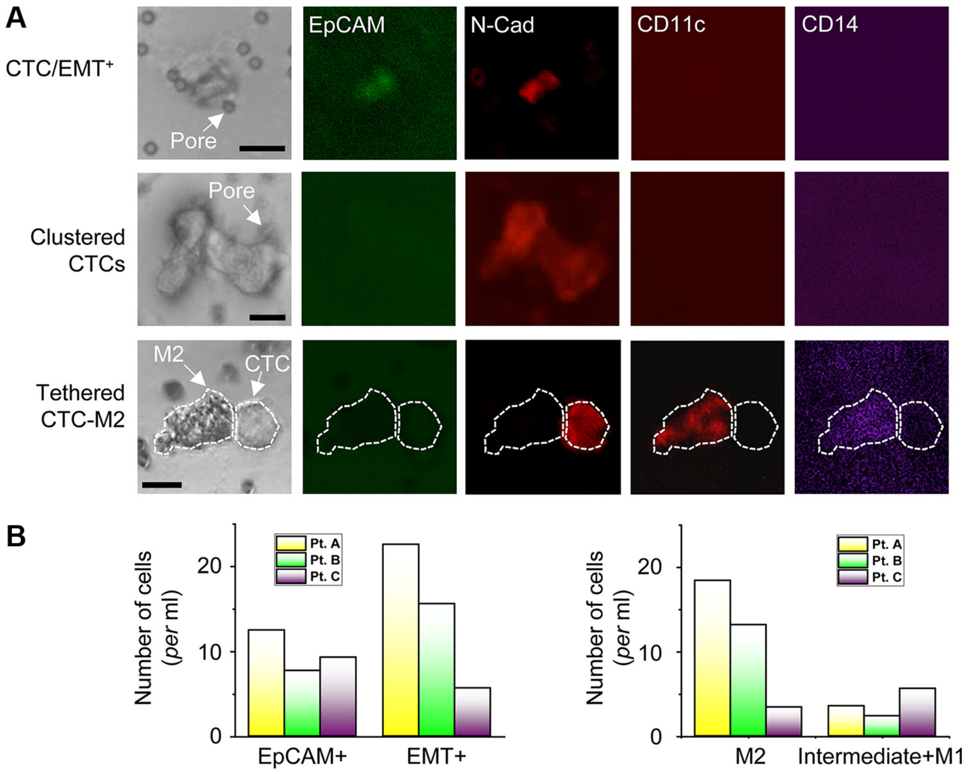

Supplementary Figure S7: Immunocytochemical analysis reveals diversity of cells isolated by microfiltration from the blood of prostate cancer patients. The cells included not only CTCs but also abundant large cells positive for immune cell markers and identified as macrophages. Some of the putative macrophages were found paired with CTCs. The macrophages were identified as M1-like/intermediate or M2-like (anti-inflammatory, potentially pro-tumor), based on their immunocytochemical profiles: staining with specific anti-CD14, CD80 and CD11c antibodies. (**A**) A gallery of images of cells retained on the filter and immunostained. Filter pores, some marked with white arrows, are well visible. The putative macrophage in lower panel was classified as M2-like (low expression of CD14, positive for CD11c). The scale bars correspond to 25 µm. (**B**) Enumeration of CTCs and macrophages from 3 individual prostate cancer patients showing the diversity of partitions of cell types.

Supplementary Table S1: Primers for qPCR. See Supplementary_Table_S1.
