# Supplementary material for: TGF-β signal rewiring sustains epithelial-mesenchymal transition of circulating tumor cells in prostate cancer xenograft hosts
Source: Oncotarget. 2016 Oct 21;7(47):77124–37. doi: 10.18632/oncotarget.12808 (PMC5363574; doi:10.18632/oncotarget.12808)
Supplement: Supplementary file 2 [file oncotarget-07-77124-s002.docx]

**Table S1. Primers for qPCR**

| **Gene symbol** | **Gene name** | **Sequence** |
| --- | --- | --- |
| *ACTB* | Actin, Beta | F: CATGTACGTTGCTATCCAGGC |
|  |  | R: CTCCTTAATGTCACGCACGAT |
| *ALDH1(A1)* | Aldehyde dehyfrogenase 1 family, member A1 | F: GCACGCCAGACTTACCTGTC |
|  |  | R: CCTCCTCAGTTGCAGGATTAAAG |
| *ASPA* | Aspartoacylase | F: GTTCCATAGCCAAGTATCCTGTG |
|  |  | R: GCGCAGGGAGGAAATTCTTTT |
| *BMP7* | Bone morphogenetic protein 7 | F: TCGGCACCCATGTTCATGC |
|  |  | R: GAGGAAATGGCTATCTTGCAGG |
| *CD133 (PROM1)* | Prominin 1 | F: AGTCGGAAACTGGCAGATAGC |
|  |  | R: GGTAGTGTTGTACTGGGCCAAT |
| *CDH1* | E-cadherin | F: CGAGAGCTACACGTTCACGG |
|  |  | R: GGGTGTCGAGGGAAAAATAGG |
| *CDH2* | N-cadherin | F: TCAGGCGTCTGTAGAGGCTT |
|  |  | R: ATGCACATCCTTCGATAAGACTG |
| *COL1A2* | Collagen, type 1, alpha 2 | F: GGCCCTCAAGGTTTCCAAGG |
|  |  | R: CACCCTGTGGTCCAACAACTC |
| *CXCL13* | Chemokine (C-X-C motif) ligand 13 | F: GCTTGAGGTGTAGATGTGTCC |
|  |  | R: CCCACGGGGCAAGATTTGAA |
| *DAB2IP* | Disabled homolog 2 interactive protein | F: GTACCGGGAGACCGACAAGA |
|  |  | R: GATGCGGATCATGGGTCCAG |
| *EEF1G* | Eukaryotic translation elongation factor 1 gamma | F: AACCGCACCCCTGAATTTCTC |
|  |  | R: GGCGTTGCTCTCAAACACAC |
| *EGFR (Her/ERBB1)* | Epidermal growth factor receptor | F: AGGCACGAGTAACAAGCTCAC |
|  |  | R: ATGAGGACATAACCAGCCACC |
| *EpCAM* | Epithelial cell adhesion molecule | F: AATCGTCAATGCCAGTGTACTT |
|  |  | R: TCTCATCGCAGTCAGGATCATAA |
| *ESR2 (ESRb)* | Estrogen receptor 2 | F: TCCATCGCCAGTTATCACATCT |
|  |  | R: CTGGACCAGTAACAGGGCTG |
| *FN1* | Fibronectin 1 | F: AGGAAGCCGAGGTTTTAACTG |
|  |  | R: AGGACGCTCATAAGTGTCACC |
| *FOXC2* | Forkhead box C2 | F: CCTCCTGGTATCTCAACCACA |
|  |  | R: GAGGGTCGAGTTCTCAATCCC |
| *FZD4* | Frizzled family receptor 4 | F: GTCTTTCAGTCAAGAGACGCTG |
|  |  | R: GTTGTGGTCGTTCTGTGGTG |
| *GAPDH* | Glyceraldehyde-3-phosphate dehydrogenase | F: ACAACTTTGGTATCGTGGAAGG |
|  |  | R: GCCATCACGCCACAGTTTC |
| *GSK3B* | Glycogen synthase kinase 3 beta | F: AGACGCTCCCTGTGATTTATGT |
|  |  | R: CCGATGGCAGATTCCAAAGG |
| *IGFBP4* | Insulin-like growth factor binding protein 4 | F: GGTGACCACCCCAACAACAG |
|  |  | R: GAATTTTGGCGAAGTGCTTCTG |
| *ITGA5* | Integrin, alpha 5 | F: GGCTTCAACTTAGACGCGGAG |
|  |  | R: TGGCTGGTATTAGCCTTGGGT |
| *ITGB1 (CD29)* | Integrin, beta 1 | F: CCTACTTCTGCACGATGTGATG |
|  |  | R: CCTTTGCTACGGTTGGTTACATT |
| *KLK4* | Kallikrein-related peptidase 4 | F: GCCAAATCATAAACGGCGAGG |
|  |  | R: CGCCCGAGCAGAACAATTC |
| *KRT7(CK7)* | Keratin 7 | F: TCCGCGAGGTCACCATTAAC |
|  |  | R: GCTCTGTCAACTCCGTCTCAT |
| *KRT8(CK8)* | Keratin 8 | F: CAGAAGTCCTACAAGGTGTCCA |
|  |  | R: CTCTGGTTGACCGTAACTGCG |
| *MMP9* | Matrix metallopeptidase 9 | F: TGTACCGCTATGGTTACACTCG |
|  |  | R: GGCAGGGACAGTTGCTTCT |
| *NOTCH1* | Notch 1 | F: GAGGCGTGGCAGACTATGC |
|  |  | R: CTTGTACTCCGTCAGCGTGA |
| *PCA-3* | Prostate cancer antigen 3 | F: TGGGAAGGACCTGATGATACA |
|  |  | R: CCCAGGGATCTCTGTGCTT |
| *PSA (KLK3)* | Prostate specific antigen or Kallikrein-related peptidase 3 | F: GTGTGTGGACCTCCATGTTATT |
|  |  | R: TGCCCCATGACGTGATACCT |
| *PTCH1* | Patched 1 | F: CCAGAAAGTATATGCACTGGCA |
|  |  | R: GTGCTCGTACATTTGCTTGGG |
| *PTCH2* | Patched 2 | F: GCTTCGTGCTTACTTCCAGGG |
|  |  | R: CATGCGGAGACCTAATGCCA |
| *PTPRC (CD45)* | Protein Tyrosine Phosphatase, Receptor Type, C | F: ACCACAAGTTTACTAACGCAAGT |
|  |  | R: TTTGAGGGGGATTCCAGGTAAT |
| *SMAD2* | SMAD family member 2 | F: CGTCCATCTTGCCATTCACG |
|  |  | R: CTCAAGCTCATCTAATCGTCCTG |
| *SNAI1* | Snail family zinc finger 1 | F: TCGGAAGCCTAACTACAGCGA |
|  |  | R: AGATGAGCATTGGCAGCGAG |
| *SNAI2* | Snail family zinc finger 2 | F: CGAACTGGACACACATACAGTG |
|  |  | R: CTGAGGATCTCTGGTTGTGGT |
| *SOX9* | SRY (sex determining region Y)-box 9 | F: AGCGAACGCACATCAAGAC |
|  |  | R: CTGTAGGCGATCTGTTGGGG |
| *TCF3* | Transcription factor 3 | F: CCGACTCCTACAGTGGGCTA |
|  |  | R: CGCTGACGTGTTCTCCTCG |
| *TGFB1* | Transforming growth factor, beta 1 | F: GGCCAGATCCTGTCCAAGC |
|  |  | R: GTGGGTTTCCACCATTAGCAC |

|  |  |  |
| --- | --- | --- |
| *TGFB2* | Transforming growth factor, beta 2 | F: CAGCACACTCGATATGGACCA |
|  |  | R: CCTCGGGCTCAGGATAGTCT |
| *TGFB3* | Transforming growth factor, beta 3 | F: ACTTGCACCACCTTGGACTTC |
|  |  | R: GGTCATCACCGTTGGCTCA |
| *TWIST1* | Twist Family BHLH Transcription Factor 1 | F: GTCCGCAGTCTTACGAGGAG |
|  |  | R: GCTTGAGGGTCTGAATCTTGCT |
| *UBB* | Ubiquitin B | F: TCAGGCGTCTGTAGAGGCTT |
|  |  | R: ATGCACATCCTTCGATAAGACTG |
| *VIM* | Vimentin | F: AGTCCACTGAGTACCGGAGAC |
|  |  | R: CATTTCACGCATCTGGCGTTC |
| *WNT11* | Wingless-type MMTV integration site family, member 11 | F: GGAGTCGGCCTTCGTGTATG |
|  |  | R: GCCCGTAGCTGAGGTTGTC |
| *WNT5A* | Wingless-Type MMTV Integration Site Family, Member 5A | F: ATTCTTGGTGGTCGCTAGGTA |
|  |  | R: CGCCTTCTCCGATGTACTGC |
| *WNT5B* | Wingless-Type MMTV Integration Site Family, Member 5B | F: CATGGCCTACATAGGGGAGG |
|  |  | R: CTGTGCTGCAATTCCACCG |
| *ZEB1* | Zinc finger E-box binding homeobox 1 | F: GATGATGAATGCGAGTCAGATGC |
|  |  | R: ACAGCAGTGTCTTGTTGTTGT |
| *ZEB2* | Zinc finger E-box binding homeobox 2 | F: CAAGAGGCGCAAACAAGCC |
|  |  | R: GGTTGGCAATACCGTCATCC |

F: forward; R: reverse.
